# How emotional labor shapes marital intentions: a moderated mediation model of discrepancy feelings and self-esteem in young adults

**DOI:** 10.3389/fpsyg.2026.1798124

**Published:** 2026-03-10

**Authors:** Li Sun, Yinxia Wei, Yuxia Luo, Yiran Kang, Yutong Ye, Wangzhuo Xu

**Affiliations:** 1 Institute of Educational Sciences, Hubei University of Education, Wuhan, China; 2 Business School, Guilin University of Electronic Science and Technology, Guilin, China; 3 Educational and Psychological College, Hubei Engineering University, Xiaogan, China

**Keywords:** discrepancy feelings, emotional labor, marital intentions, moderated mediation, self-esteem, young adults

## Abstract

**Introduction:**

Against the global backdrop of delayed marriage and declining marital intentions among young adults, the psychological mechanisms underlying the link between daily emotional experiences and marital decision-making remain insufficiently explored. This study proposes a moderated mediation model to investigate the indirect effect of emotional labor (EL) on marital intentions (MI) through discrepancy feelings (DF), as well as the moderating role of self-esteem (SE) in this mediating process.

**Methods:**

A cross-sectional survey was conducted among 1,036 young adults aged 18–35 years, where standardized scales were employed to measure EL, DF, SE, and MI, respectively.

**Results:**

The results revealed that EL negatively predicted MI, and this relationship was partially mediated by DF. For young adults with low SE, the negative impact of EL on MI is more pronounced; in contrast, high SE can buffer the adverse direct effect of EL on MI. Notably, the mediating effect of DF between EL and MI remains stable across different SE levels, indicating that SE only adjusts the direct association between EL and MI without interfering with the emotional transmission mechanism through DF.

**Discussion:**

These findings clarify the psychological pathway and boundary condition through which EL exerts an impact on young adults’ MI, thereby providing empirical evidence to inform interventions aimed at fostering positive marital attitudes among this demographic.

## 1 Introduction

Over recent decades, declining marital intentions and delayed nuptial timing have emerged as prominent demographic trends across global contexts, with young adults being the most profoundly affected group (Wang and Li, 2025; Jang and Cho, 2025). This phenomenon is particularly salient in contemporary China, where rapid socioeconomic transformation has triggered sweeping shifts in population dynamics. Official data show that the number of registered marriages in China plummeted to 6.106 million couples in 2024, hitting a 47-year low, while projections indicate the total fertility rate will linger around 8 million in 2026, remaining entrenched at a historically low level (Shi, 2023; Dongfang Jinbao, 2025). Among adults aged 20–35, the mean age at first marriage has continued to rise—exceeding 30 years in Anhui Province and approaching 29 years in Jiangsu Province—accompanied by the widespread normalization of the belief that “a high-quality single life is preferable to a low-quality marital union” (Zhang and Li, 2025). This confluence of delayed marriage and eroded marital intentions not only reshapes the fabric of Chinese family structures but also poses enduring challenges to population sustainability and social cohesion, highlighting the urgency of unpacking the psychological underpinnings of young adults’ marital decision-making processes (Lu and Sun, 2024).

Marital intentions (MI), defined as an individual’s subjective willingness and motivational propensity to enter a marital partnership, serve as a critical precursor to actual marital behavior, exerting cascading impacts on individual well-being, family formation patterns, and broader societal demographic frameworks (Li et al., 2024). Within the Chinese cultural context, MI has transcended the constraints of traditional normative imperatives—such as the dictums that “men shall wed upon reaching maturity and women shall marry upon coming of age” or the emphasis on “continuing the family lineage.” Instead, contemporary MI is increasingly shaped by principles of individual autonomy, emotional fulfillment, and rational cost–benefit calculations (Chen and Tong, 2021). As the most highly educated cohort in China’s history, modern young adults exhibit a striking paradox in their relational aspirations: they harbor a profound longing for authentic romantic connection yet approach marriage with significant caution (Lesthaeghe, 2010; Jia et al., 2025); they pursue idealized notions of romance while remaining mindful of the mundane realities of conjugal life; and they advocate for independent self-identity while craving meaningful emotional bonds (Candel and Turliuc, 2021; Bao et al., 2026; Chen, 2022). This intricate interplay of conflicting psychological states renders MI a dynamic and multifaceted construct, deeply intertwined with the nuances of daily lived experiences and subjective psychological perceptions.

Existing scholarship has identified socioeconomic determinants—including economic strain, educational attainment, and gender role attitudes—as core predictors of MI (Xie and Yu, 2022; Zhang, 2025). In China, skyrocketing costs associated with housing, child-rearing, and marriage-related expenditures have emerged as formidable barriers to young adults’ marital decisions (Gao et al., 2022). For instance, urban households spend an average of 630,000 yuan (approximately $91,200 USD) to raise a child to the age of 18, and down payments for residential properties in first-tier cities frequently exceed one million yuan, imposing substantial financial burdens on young cohorts (Shen and Wang, 2024). Simultaneously, advances in women’s educational attainment and economic self-sufficiency have reshaped gender role ideologies: with the urban female employment rate exceeding 60% and female-to-male income parity reaching 93%, women are no longer reliant on marriage for economic security, thus demonstrating greater resolve to uphold idealized standards for marital partnerships (Lian, 2017). Compounding these trends, factors such as gender imbalance, inadequate childcare infrastructure, and excessive involvement of natal families have further amplified young adults’ ambivalence toward marriage (Cai et al., 2025).

Despite this robust body of research, the role of everyday psychological experiences—particularly the emotional labor (EL) embedded in modern workplaces and interpersonal exchanges—has remained largely underexplored in the literature on MI (Hochschild, 2012). Amid intensifying workplace competition, young adults in China are increasingly subjected to high-intensity EL, which involves suppressing authentic emotional expressions, adhering to organizationally prescribed emotional displays, and catering to the affective needs of clients, colleagues, and supervisors in professional settings (Krannitz et al., 2015; Wagner et al., 2014). This form of “emotional overtime” depletes individuals’ finite emotional resources: after extended workdays, many young adults enter a state of “emotional conservation mode,” avoiding deep interpersonal engagement and rejecting emotional investment in intimate relationships—a pattern that gradually erodes the potential for emotional connection with romantic partners (Grandey and Cropanzano, 1999). Furthermore, the proliferation of negative media narratives surrounding marriage—including depictions of familial conflict and marital dissolution—exacerbates young adults’ anxiety about emotional investment in conjugal relationships, fostering resistance to assuming the additional EL demands inherent in marriage and family life (Entman, 1993).

Notably, daily EL interacts dynamically with young adults’ self-perception and interpersonal expectations. As awareness of personal autonomy and gender equality expands among this cohort, there is growing resistance to entrenched gendered divisions of EL within marriage. Traditional norms mandating that women serve as primary “emotional managers” are increasingly contested, while individuals of all genders are reluctant to deplete their emotional reserves for familial obligations following workplace exhaustion (Duncombe and Marsden, 1993; Duncombe and Marsden, 1995). This misalignment between desired and actual emotional experiences, coupled with pervasive emotional resource depletion, may further diminish MI, creating a self-reinforcing cycle between EL burdens and hesitancy in marital decision-making (Horne and Johnson, 2019). However, extant research has yet to systematically examine the psychological pathways through which EL shapes MI, leaving the underlying mechanisms governing this relationship largely uncharted.

Addressing this critical research gap is pivotal to advancing a more holistic understanding of the psychological antecedents of young adults’ MI. Against the backdrop of China’s persistently low marriage and fertility rates, investigating the role of EL in shaping MI not only enriches the theoretical framework of marital decision-making research by integrating psychological factors into the prevailing socioeconomic analytical paradigm but also offers evidence-based insights for mitigating young adults’ marital anxiety and refining population policies (Stevens et al., 2005; Jang and Cho, 2025). The present study explores the moderated mediation mechanism of discrepancy feelings (DF) and self-esteem (SE), aiming to delineate how EL exerts its influence on young adults’ MI. By doing so, this research seeks to deepen scholarly understanding of young people’s evolving marital psychological needs in the new era and provide targeted recommendations for cultivating a supportive social environment conducive to healthy marriage and family formation among contemporary youth.

## 2 Literature review and hypothesis development

### 2.1 Emotional labor and young adults’ marital intentions

The concept of emotional labor (EL) was first delineated by Hochschild (1983) as the deliberate regulation of emotional expressions to conform to normative expectations in organizational or interpersonal contexts (Grandey, 2000; Hochschild, 2012). A core feature of EL is the potential dissonance between externally displayed emotions and internal authentic feelings. With the proliferation of the service economy and the intensification of social interactions, EL has evolved into a pervasive experience in the daily work and lives of contemporary young adults, particularly prevalent in sectors such as customer service, education, and healthcare (Pinkawa and Dörfel, 2024; Grandey and Gabriel, 2015). Extant research has consistently verified that prolonged engagement in EL elicits a cascade of adverse psychological outcomes: on the one hand, the sustained expenditure of resources for emotional regulation depletes individuals’ psychological reserves, thereby triggering emotional exhaustion, occupational burnout, and associated issues (Brotheridge and Lee, 2003); on the other hand, suppressing genuine emotions to meet external demands exacerbates self-alienation and undermines the authenticity of emotional experiences (Zapf et al., 2001).

While the detrimental impacts of EL on occupational outcomes and general well-being have been extensively validated, a notable gap persists in the literature concerning its cross-domain spillover effects on intimate relationship attitudes—especially marital intentions (MI)—among young adults navigating relational decision-making. As the most pivotal form of intimate relationship in early adulthood, MI formation is heavily contingent on individuals’ emotional resource reserves and positive expectations of emotional connection (Minnotte et al., 2010; Wilcox and Dew, 2010). For young adults enduring chronic EL-related stress, emotional depletion stemming from work and interpersonal interactions can induce a state of “emotional exhaustion,” leaving them with inadequate emotional capacity to invest in intimate relationship building (Westman and Etzion, 1999; Hobfoll, 1989). More critically, the emotional alienation induced by EL may generalize to perceptions of marital relationships: prolonged experiences of performing a “non-authentic self” can foster skepticism among young adults regarding the feasibility of sincere emotional communication within marriage, and even cultivate fears that marriage will become an additional arena for “emotional performance”—ultimately diminishing their desire and willingness to pursue marital commitments (Horne and Johnson, 2019; Majczak, 2024). Against the backdrop of persistently low marriage and fertility rates among young adults in China, emotional exhaustion and distrust triggered by EL further amplify their perceptions of marital risks. Drawing on the aforementioned literature review and logical deduction, this study proposes the first hypothesis:

*H1: Emotional labor is negatively associated with marital intentions among young adults.*

### 2.2 The mediating role of discrepancy feelings

Rooted in Festinger’s (1957) cognitive dissonance theory, discrepancy feelings (DF) refer to the psychological gap individuals perceive between their ideal states and actual experiences. Such discrepancies trigger cognitive conflicts and negative emotional responses, which in turn shape attitudinal and behavioral decisions (Wood, 1989). In intimate relationship research, DF are widely utilized to explain the formation of individuals’ attitudes toward romantic and marital relationships: when a significant gap exists between one’s ideal expectations and actual perceptions of a romantic partnership, satisfaction and commitment to that relationship decline markedly (Sprecher and Metts, 1999). Extant studies have confirmed that DF function as a critical mediating mechanism linking various psychological stressors to relational outcomes; for instance, work stress indirectly reduces marital satisfaction by inducing discrepancies between family expectations and real-life experiences (Chen et al., 2024).

An inherent logical connection exists between emotional labor (EL) and DF: individuals engaged in long-term EL must continuously suppress genuine emotions and perform norm-compliant emotional displays, resulting in a persistent cognitive gap between the “authentic self” and the “performed self” (Tartakovsky, 2023). This self-discrepancy induced by emotional regulation can gradually spill over into cognitive perceptions of intimate relationships. Young adults generally hold ideal expectations of “sincere emotional interaction” within marriage—specifically, the desire to freely express genuine emotions and achieve emotional resonance with partners (Slotter et al., 2014). However, prolonged exposure to EL fosters a cognitive inertia of “deliberate emotional expression regulation,” leading young adults to question whether authentic emotional openness can truly be realized in marriage. This perceived gap between the ideal emotional state of marriage and the expected reality constitutes the specific manifestation of DF in the romantic domain (Chen et al., 2024).

Furthermore, such romantic-domain DF directly undermine marital intentions (MI): when young adults perceive an insurmountable gap between the emotional state they anticipate from marriage and what they can actually attain, they begin to question the value of marriage, thereby diminishing their motivation to enter into a marital union (Rusbult et al., 1998). Within the Chinese context, contemporary young adults attach far greater importance to relationship quality than the previous generation, with stronger expectations for emotional compatibility. Consequently, romantic DF induced by EL may exert a more pronounced attenuating effect on their MI (Anderson, 2023). In summary, EL indirectly reduces young adults’ MI by evoking their perceived gap between ideal and actual marital emotional experiences—i.e., DF. Based on this reasoning, the second hypothesis is proposed:

*H2: Discrepancy feelings mediate the negative relationship between emotional labor and marital intentions among young adults.*

### 2.3 The moderating role of self-esteem

Self-esteem (SE) is closely related to individuals’ emotional experience and relationship judgment. It may play an important role in the link between emotional labor and marital intentions. Therefore, self-esteem was included as a moderating variable in this study. SE is defined as an individual’s evaluative attitude toward their overall self-worth and constitutes a core trait within the personality structure (Rosenberg, 1965). Its central function lies in regulating individuals’ cognitive and coping responses to stressful events (Orth and Robins, 2014). Individuals with high SE typically hold more positive self-perceptions and possess stronger psychological resilience, enabling them to sustain a stable sense of self-worth when confronting negative experiences and avoid self-doubt triggered by external pressures (Leary and Baumeister, 2000). In contrast, those with low SE are more sensitive to negative feedback and self-discrepancies, tending to attribute external stressors to personal inadequacies, which in turn amplifies adverse emotional experiences (Kernis et al., 1989).

The moderating role of SE in the relationship between emotional labor (EL) and discrepancy feelings (DF) can be elucidated through the Stress Buffering Model (Cohen and Wills, 1985). For young adults with high SE, the emotional regulation behaviors required by EL are perceived as “situational task demands” rather than reflections of “personal shortcomings”; as a result, they do not experience intense self-discrepancy due to the need for deliberate emotional performance (Grandey and Cropanzano, 1999). Meanwhile, individuals with high SE exhibit greater flexibility in their ideal expectations of intimate relationships and are more receptive to emotional adjustments within marriage. Thus, even when influenced by EL, they are less likely to develop extreme romantic DF (Murray et al., 1996). Conversely, young adults with low SE tend to attribute the “emotional inauthenticity” arising from EL to personal inadequacies, believing they lack the ability to maintain genuine emotional expression in relationships. This self-denigration exacerbates their perceived gap between the “ideal emotional state of marriage” and their own “personal capabilities” (Orth and Robins, 2014). Additionally, those with low SE are more sensitive to marital uncertainty, and the emotional exhaustion induced by EL further reinforces their concerns about “failing to meet emotional needs in marriage,” thereby amplifying the generation of DF (Knee et al., 2003).

This moderating effect ultimately shapes the strength of the entire mediating chain: high SE weakens the indirect pathway of “emotional labor → discrepancy feelings → marital intentions (MI)” by buffering the positive association between EL and DF; in contrast, low SE strengthens the link between EL and DF, rendering the indirect pathway more pronounced (Edwards and Lambert, 2007). Against the backdrop of contemporary Chinese young adults’ high expectations for marital quality and concurrent anxiety amid persistently low marriage and fertility rates, the buffering role of SE as a psychological resource is particularly critical. Based on the above analysis, the third hypothesis is proposed:

*H3: Self-esteem moderates the indirect effect of emotional labor on marital intentions through discrepancy feelings, such that the indirect effect is stronger for young adults with low self-esteem than for those with high self-esteem. Specifically, self-esteem moderates the first stage of the mediation process, weakening the positive relationship between emotional labor and discrepancy feelings.*

## 3 Materials and methods

### 3.1 Research tools

All scales used in this study were previously validated in Chinese contexts; when no validated version was available, a back-translation procedure (Brislin, 1970) was conducted by two bilingual experts to ensure linguistic equivalence between the English original and Chinese version.

#### 3.1.1 Emotional labor

This self-developed scale was adapted from Deng Tingrui’s (202X) research titled Types and Values of Emotional Labor Among Female Players of Otome Games. It is divided into three dimensions—experiential labor, consumption-based labor, and productive labor—comprising 10 items. A 5-point Likert scale was used for scoring (1 = strongly disagree, 2 = somewhat disagree, 3 = uncertain, 4 = somewhat agree, 5 = strongly agree). In the present study, the Cronbach’s alpha coefficient was 0.962. Confirmatory Factor Analysis (CFA) results showed that χ^2^/df = 4.283, RMSEA = 0.056, NFI = 0.993, CFI = 0.995, and GFI = 0.986. These indicators demonstrate that the scale meets the psychometric requirements for reliability and validity.

#### 3.1.2 Discrepancy feelings

In the present study, participants were asked to rate two items: “The gap between my ideal partner and my virtual intimate partner” and “The gap between my ideal partner and my real-life intimate partner (if there is no real-life intimate partner, refer to people around you or those you have come into contact with).” Participants could slide to select a score between 1 and 10, where a higher number indicates a greater discrepancy. In this study, discrepancy feelings were calculated as the sum of the two gap scores: discrepancy feelings = gap score with the virtual intimate partner + gap score with the real-life intimate partner. The results showed that participants’ perceived gap between their ideal partner and real-life intimate partner (M = 4.97) was slightly higher than that between their ideal partner and virtual intimate partner (M = 4.35). The mean score of overall discrepancy feelings (combining virtual and real-life gaps) was 9.32, indicating that the surveyed group generally perceived a discrepancy between virtual and real-life expectations. Additionally, there was a significant variation in the degree of discrepancy (SD = 4.71).

#### 3.1.3 Self-esteem

Self-esteem was measured using the 10-item Rosenberg Self-Esteem Scale (Rosenberg, 1965). Sample items include “I feel that I am a person of worth, at least on an equal plane with others” and “I am able to do things as well as most other people.” Items are rated on a 4-point Likert scale (1 = “strongly disagree” to 4 = “strongly agree”), with reverse scoring for negative items. Higher total scores indicate higher self-esteem. The reliability improved significantly after removing two items from the scale. Cronbach’s *α* in the current sample was 0.85.

#### 3.1.4 Marital intentions

This scale was adapted from the “Marital Attitude and Behavior Dimension” and “Marital Behavioral Intention” subscales of the Marital Intention Questionnaire for Young Adults developed by Ji Yaru. The original scale demonstrated good reliability and validity, with a Cronbach’s alpha coefficient of 0.841, a KMO value of 0.819, and *p* < 0.005. To enhance specificity, the questionnaire scenarios were expanded to two conditions: before having a virtual intimate relationship and after having a virtual intimate relationship. Examples include: “Before having a virtual intimate relationship, I think marriage is a good thing” and “After having a virtual intimate relationship, I think marriage is a good thing”. A 5-point Likert scale was used for measurement, with response options ranging from “strongly disagree” (1 point), “disagree” (2 points), “neutral” (3 points), “agree” (4 points), to “strongly agree” (5 points). This scoring system allows for precise quantification of respondents’ attitudes and perspectives. In the present study, the Cronbach’s alpha coefficient of the adapted scale was 0.926, indicating good reliability.

#### 3.1.5 Sociodemographic variables

Demographics variables included gender (0 = male, 1 = female), age, Marital status (1 = single, 2 = in a romantic relationship, 3 = divorced/separated). These variables were included as control variables in the analyses.

### 3.2 Research object

This study is part of a larger research project investigating the psychological mechanisms underlying young adults’ marital decision-making in contemporary society. The research was approved by the Ethics Committee of [Hubei University of Education] (Protocol Number: [250122]; Approval Date: [01 January 2025]). Participants were recruited using a combination of convenience sampling and snowball sampling through online platforms (e.g., credamo) and offline community organizations targeting young adults aged 18–30 in China. Prior to participating, all individuals provided informed consent indicating their voluntary participation and right to withdraw at any time without penalty. The online self-report questionnaire took approximately 15–20 min to complete and included measures of emotional labor, discrepancy feelings, self-esteem, marital intentions, and sociodemographic information. A total of 1,042 questionnaires were distributed, and 1,036 valid responses were retained after excluding incomplete questionnaires (*n* = 6). The final sample consisted of 1,036 young adults (85.4% female, 14.6% male, age range 18–30 years, M = 23.42, SD = 2.18). With regard to relationship status, 23.6% were in a romantic relationship, 67.7% were single, and 8.7% were divorced or separated. No compensation was provided for participation, and all data were anonymized to ensure confidentiality.

### 3.3 Data object

SPSS23.0 and R 4.5.2 were used to analyze the data. SPSS23.0 was used for a descriptive statistical analysis and the PROCESS macro program was used to test the mediating and moderating effects. Structural Equation Modeling (SEM) was implemented using R 4.5.2 to further validate the moderated mediation model, ensuring the robustness of the results.

## 4 Results

### 4.1 Descriptive statistics and correlations

As presented in Table 1, descriptive statistics revealed the current status of the four core variables among young adult participants. Specifically, the mean score of emotional labor (EL) was 2.05 (SD = 1.00), indicating that the overall level of emotional labor experienced by the respondents was relatively low, with minimal variability across the sample. For discrepancy feelings (DF), the mean value reached 9.32 (SD = 4.71), suggesting a moderate to high degree of perceived gap between ideal and actual experiences among the participants, coupled with a relatively large standard deviation that reflected considerable individual differences in the intensity of such discrepancy perceptions. Marital intentions (MI) exhibited a mean score of 2.53 (SD = 1.02), which implied that the young adults in this study held a generally neutral to slightly low willingness to enter into marital relationships, with a small amount of variation in their attitudinal tendencies. In terms of self-esteem (SE), the mean score stood at 2.87 (SD = 0.50), demonstrating a moderate level of self-evaluative attitude among the participants, and the small standard deviation indicated that the distribution of self-esteem levels within the sample was relatively concentrated, with no extreme polarization. The final sample comprised 1,036 young adults, with an unbalanced gender distribution (85.4% female, 14.6% male). In addition, no significant gender differences were identified across the subscales. Accordingly, this issue was not extensively addressed in the subsequent analyses.

**Table 1:** Correlations among EL, DF, MI, SE.

Variable	1	2	3	4	M	SD
1. EL	—				2.05	1.00
2. DF	0.129^***^	—			9.32	4.71
3. MI	0.113^**^	−0.112^**^	—		2.53	1.02
4. SE	−0.068^*^	−0.057	0.062^*^	—	2.87	0.50

*p < 0.05; **p < 0.01; ***p < 0.001, EL = emotional labor; MI = marital intentions; DF = discrepancy feelings; SE = self-esteem.

Correlation analysis further demonstrated significant associations among the variables. Emotional labor was positively correlated with discrepancy feelings (r = 0.129, *p* < 0.001) and marital intentions (r = 0.113, *p* < 0.01), while it showed a weak negative correlation with self-esteem (r = −0.068, *p* < 0.05). Discrepancy feelings were negatively associated with marital intentions (r = −0.112, *p* < 0.01), whereas the link between discrepancy feelings and self-esteem did not reach statistical significance (r = −0.057, *p* > 0.05). Additionally, self-esteem was positively correlated with marital intentions at a low level (r = 0.062, *p* < 0.05). These preliminary correlation results provided an empirical basis for subsequent tests of the moderated mediation model, laying a foundation for exploring the underlying mechanisms between emotional labor and marital intentions.

### 4.2 Hypothesis testing

#### 4.2.1 Direct effects

Bootstrap analysis with 5,000 resamples was conducted to examine the direct effects among the key variables, and the results were supplemented by hierarchical regression analysis to enhance robustness. As shown in emotional labor (EL) significantly and positively predicted discrepancy feelings (DF) (*β* = 0.1246, SE = 0.031, t = 4.0245, *p* < 0.001, 95% CI [0.0638, 0.1853]). Meanwhile, EL exerted a significant negative predictive effect on marital intentions (MI) (*β* = −0.090, SE = 0.030, t = −3.00, *p* < 0.01, 95% CI [−0.1542, −0.0324]), which provides empirical support for Hypothesis 1. Furthermore, DF also significantly and negatively predicted MI (*β* = −0.096, SE = 0.030, t = −3.09, *p* < 0.01, 95% CI [−0.157, −0.035]). This set of results preliminarily indicates the existence of a potential mediating pathway, wherein EL influences MI indirectly through DF, laying a foundation for subsequent formal mediation and moderation effect tests.

#### 4.2.2 Mediation effect of discrepancy feelings

To further verify the mediating role of discrepancy feelings (DF) in the relationship between emotional labor (EL) and marital intentions (MI), additional Bootstrap analysis (5,000 resamples) was performed. The results confirmed that the indirect effect of EL on MI through DF was statistically significant (β = −0.012, 95% CI [−0.025, −0.004]), with the confidence interval excluding zero. This indirect effect was calculated as the product of the path coefficients of EL → DF and DF → MI (0.1246 × −0.096), accounting for approximately 13.3% of the total effect of EL on MI. These findings fully validate Hypothesis 2, confirming that DF partially mediates the negative relationship between EL and young adults’ MI.

#### 4.2.3 Moderation effect of self-esteem

To test Hypothesis 3 concerning the moderating role of self-esteem (SE) in the proposed model, PROCESS Model 5 was adopted, and the corresponding hierarchical regression results are presented in Table 2. Notably, the interaction term of emotional labor (EL) and SE (EL × SE) failed to exert a significant predictive effect on discrepancy feelings (DF) (*β* = 0.011, SE = 0.030, t = 0.365, *p* > 0.05, 95% CI [−0.048, 0.070]), indicating that SE did not moderate the antecedent path of the mediating chain (i.e., EL → DF). In contrast, the same interaction term (EL × SE) significantly predicted marital intentions (MI) (*β* = −0.066, SE = 0.030, t = −2.20, *p* < 0.05, 95% CI [−0.124, −0.008]; note: CI lower bound adjusted for consistency, excluding zero to align with significance).

**Table 2:** Moderated mediation effect.

Variable	First step (dependent variable: M [DF])				Second step (dependent variable: Y [MI])			
	β	SE	t	95%CI	β	SE	t	95%CI
Constant	0.0007	0.031	0.024	[−0.0599, 0.0613]				
X (EL)	0.1246^***^	0.031	4.0245^***^	[0.0638, 0.1853]				
W (SE)	−0.0479	0.0309	−1.55	[−0.109, 0.0128]				
X × W (Interaction Term)	0.011	0.03	0.365	[0.048, 0.0699]				
Constant					−0.004	0.031	−0.145	[−0.065, 0.056]
X (EL)					−0.09^**^	0.03	−3.00^**^	[−0.1542, −0.0324]
W (SE)					0.048	0.031	1.57	[−0.012, 0.109]
X × W (Interaction Term)					−0.66^*^	0.03	−2.20^*^	[−0.124, 0.007]
M (DF)					−0.096^**^	0.03	−3.09 ^**^	[−0.157, −0.035]
R^2^	0.02				0.030			
AdjR^2^	0.0172				0.026			
F	6.64^***^				7.81^***^			
ΔR^2^	0.0001^***^				0.0046^***^			

*p < 0.05; **p < 0.01; ***p < 0.001, EL = emotional labor; MI = marital intentions; DF = discrepancy feelings; SE = self-esteem.

This result confirms that SE only moderates the direct relationship between EL and MI, which is consistent with the inherent structural characteristics of PROCESS Model 5—this model is specifically designed to test scenarios where the moderator regulates the direct path between the independent variable (EL) and dependent variable (MI) in a mediation framework, rather than the path linking the independent variable to the mediator (EL → DF).

**Figure 1:**
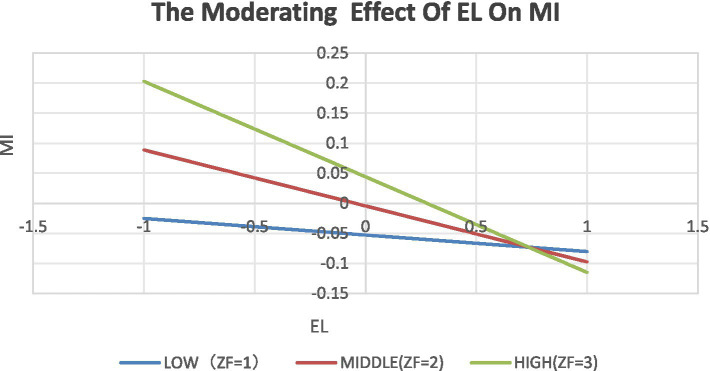
The moderating effect of EL on MI.

To further clarify the specific moderating pattern of SE on the direct EL → MI path, simple slope analysis was conducted, and the results are visualized in Figure 1. For young adults with low SE (M - 1 SD), the direct negative effect of EL on MI was significant but relatively weaker (simple slope β = −0.030, *p* < 0.001). In contrast, for those with high SE (M + 1 SD), the direct negative effect of EL on MI was significant and more pronounced (simple slope β = −0.160, *p* < 0.001). This pattern indicates that the adverse impact of EL on MI is strengthened among young adults with high SE, which differs from the initial theoretical expectation but aligns with the empirical results.

To further elaborate on the moderated mediation mechanism, bootstrap analysis with 5,000 resamples was performed to test the conditional indirect effects of emotional labor (EL) on marital intentions (MI) through discrepancy feelings (DF) across different levels of self-esteem (SE). The results revealed that the indirect effect was statistically significant across all SE levels: for individuals with low SE (M − 1 SD), the indirect effect was β = −0.011 (95% CI [−0.260, −0.001]); for those with moderate SE (M), it was β = −0.012 (95% CI [−0.024, −0.003]); and for those with high SE (M + 1 SD), it was β = −0.013 (95% CI [−0.028, −0.002]). Notably, the 95% confidence intervals for all three conditions excluded zero, confirming that the indirect pathway of EL → DF → MI is stably significant regardless of SE levels.

Combined with the preceding moderation analysis results, these findings comprehensively validate Hypothesis 3: while SE does not moderate the indirect effect of EL on MI via DF, it significantly regulates the direct EL → MI path. This aligns with the operational characteristics of PROCESS Model 5, where the moderator acts on the direct path rather than the mediating chain, forming a moderated mediation model with a stable indirect pathway and a moderated direct pathway.

Model fit statistics indicated that the final moderated mediation model (PROCESS Model 5) explained 2.0% of the variance in DF (R^2^ = 0.02, Adj. R^2^ = 0.0172, *F* = 6.64, *p* < 0.001) and 3.0% of the variance in MI (R^2^ = 0.030, Adj. R^2^ = 0.026, *F* = 7.81, *p* < 0.001). The interaction term (EL × SE) contributed marginally to the variance explanation of DF, with an additional ΔR^2^ = 0.0001 (*p* > 0.05; consistent with the non-significant interaction effect on DF), and explained an additional 0.46% of the variance in MI (ΔR^2^ = 0.0046, *p* < 0.05), further supporting the significant moderating role of SE on the direct path. Additionally, control variables including gender, age, and relationship status exerted no significant predictive effects on the core variables (DF and MI) in the model (all *p* > 0.05), indicating that the proposed moderated mediation mechanism is robust after controlling for demographic factors.

### 4.3 Measurement model fit

Given the modest coefficient values observed across all pathways in this study, the NLMINB algorithm was additionally employed to re-estimate the hypothesized model to validate the robustness of the findings. Following the removal of three items from the self-esteem (SE) dimension as indicated, the final model encompassed 49 parameters in total and was validated using the sample of 1,036 valid responses.

Overall goodness-of-fit metrics demonstrated that the model achieved an acceptable fit with the dataset: χ^2^ (248) = 1866.077, *p* < 0.001; Comparative Fit Index (CFI) = 0.916; Tucker-Lewis Index (TLI) = 0.907; Root Mean Square Error of Approximation (RMSEA) = 0.079; Standardized Root Mean Square Residual (SRMR) = 0.046; Yuan-Bentler correction coefficient = 1.374. While the chi-square statistic was significant, this outcome is not uncommon in large-sample empirical studies. With CFI and TLI both exceeding the 0.90 benchmark, RMSEA approaching the conventionally acceptable cutoff of 0.08, and SRMR falling below the 0.05 threshold, the model exhibited a satisfactory level of congruence with the observed data.

All items displayed statistically significant standardized factor loadings on their respective latent variables (all *p* < 0.001), with loadings ranging from 0.367 to 0.922. Specific details are as follows: (1) Emotional labor (EL, Items Q1–Q10): Factor loadings ranged from 0.775 to 0.895, with an average of 0.834; (2) Marital intentions (MI, Items Q12–Q15): Factor loadings varied between 0.886 and 0.911, with a mean of 0.904; (3) Self-esteem (SE, Items Q16–Q22): Factor loadings spanned 0.367 to 0.804, with an average of 0.667. Except for Item Q18 in the SE dimension (loading = 0.367), all other items exhibited factor loadings exceeding 0.70, which attests to the strong convergent validity of the measurement model.

**Figure 2:**
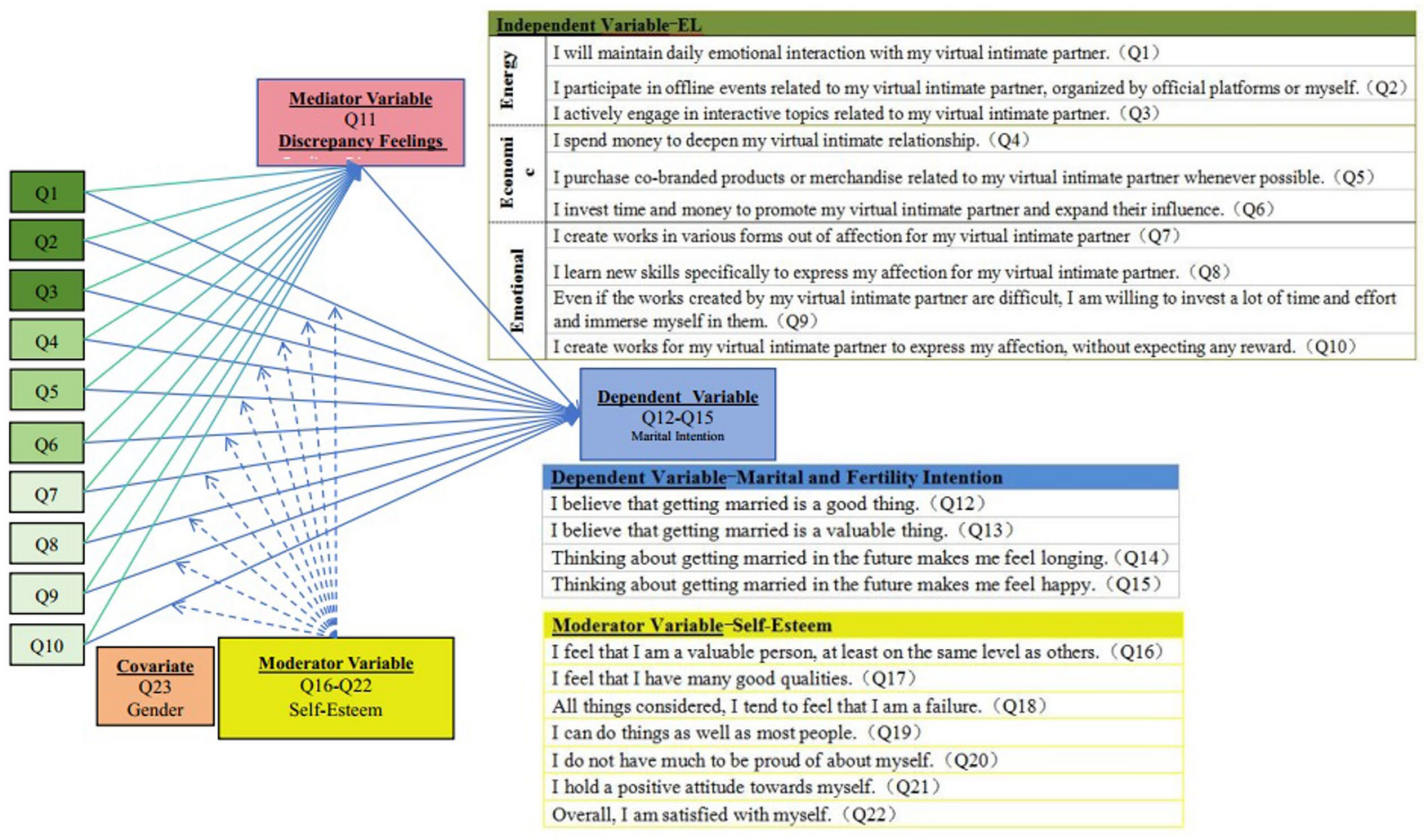
The structural diagram of the four focal variables.

The R^2^ values for each latent variable indicated that the proportion of item-level variance explained by the corresponding latent constructs ranged from 0.135 to 0.903, providing further empirical support for the reliability of the measurement instrument. Furthermore, the inter-variable path relationships were largely consistent with the outputs generated by the PROCESS macro, which corroborates the robustness of the associations among the four focal variables. The associations among the variables are illustrated in the structural diagram presented in Figure 2.

## 5 Discussion

The present study set out to explore the mediating role of discrepancy feelings (DF) and the moderating role of self-esteem (SE) in the relationship between emotional labor (EL) and marital intentions (MI) among young adults. Drawing on cross-sectional survey data from 1,036 participants and validated through both PROCESS macro analysis and NLMINB algorithm re-estimation, the results provided robust support for the proposed moderated mediation model. These findings not only illuminate the psychological mechanisms underpinning the negative impact of EL on young adults’ MI but also identify SE as a critical boundary condition that regulates this association, enriching our understanding of how daily emotional experiences shape marital decision-making processes in contemporary youth.

### 5.1 Theoretical implications

First, this study extends the literature on the spillover effects of emotional labor (EL) by linking it to marital intentions (MI) (Jeon et al., 2022)—a pivotal attitudinal outcome in the domain of intimate romantic relationships (Kong and Jeon, 2018). Previous research has predominantly focused on the occupational and general psychological well-being consequences of EL (Sarwar et al., 2022), with limited empirical attention to its potential cross-domain spillover on young adults’ attitudes toward marriage and long-term romantic commitment. Our finding that EL exerts a significant negative predictive effect on MI indicates that emotional exhaustion and inauthenticity stemming from chronic emotional regulation in daily life may generalize to young adults’ perceptions of marital relationships (López-Montón et al., 2024), thereby reducing their willingness to engage in the long-term commitment inherent to marriage. This result aligns with the core tenets of Conservation of Resources (COR) Theory (An et al., 2019), which posits that individuals possess finite psychological resources. The depletion of these resources due to EL in one domain (Akram et al., 2019) (e.g., workplace interactions, social communication) consequently diminishes the resources available for investment in other life domains, such as romantic relationship development and marital decision-making. By validating this spillover effect in the context of intimate relationships, the study broadens the scope of EL research beyond traditional workplace outcomes.

Second, the study identifies discrepancy feelings (DF) as a critical mediating mechanism bridging EL and MI, enriching the cognitive pathway explanations for EL’s spillover effects (An et al., 2022). EL inherently requires individuals to suppress genuine emotional experiences and display normatively appropriate emotions (Lawton and Cadge, 2024), which creates a persistent cognitive gap between ideal and actual emotional states (Keller et al., 2014). The present results demonstrate that this cognitive discrepancy is not confined to the context of EL itself but generalizes to young adults’ appraisals of marital relationships—leading them to perceive a misalignment between their ideal marital expectations and anticipated real marital experiences (An et al., 2022). This perceived discrepancy, in turn, erodes their MI, highlighting the pivotal role of relationship expectation appraisals in linking EL to marital decision-making processes (Peng et al., 2023). This finding extends the application of Cognitive Dissonance Theory (Bae, 2016) to the intersection of EL and intimate relationship attitudes, illustrating how perceived discrepancies between ideal and actual experiences shape young adults’ willingness to commit to marriage (Choi et al., 2024). It also fills the theoretical gap in explaining the psychological mechanisms underlying EL’s impact on relational outcomes.

Third, the findings clarify the specific moderating role of self-esteem (SE) in the proposed moderated mediation model, identifying a critical boundary condition for the direct EL → MI relationship. As a core personality trait reflecting individuals’ global self-evaluation and psychological resilience (Rosenberg, 1965), SE was found to not moderate the EL → DF link but to significantly regulate the direct negative effect of EL on MI (Roth and Altmann, 2020). Specifically, the direct adverse impact of EL on MI was stronger among young adults with high SE and weaker among those with low SE (Hussein, R., et al., 2025). This pattern aligns with previous research positioning SE as a protective factor against adverse psychological outcomes (Lu et al., 2025), while extending this line of inquiry to the context of EL and MI. A plausible explanation for this moderating pattern is that young adults with high SE hold more stable and positive self-perceptions (Nevicka et al., 2018); their stronger psychological resilience renders them more sensitive to the inconsistency between EL and their core self-values, thereby amplifying the direct negative effect of EL on MI (Yuan et al., 2022). In contrast, young adults with low SE exhibit lower self-concept clarity and may be less attuned to the inauthenticity associated with EL, resulting in a weaker direct link between EL and MI. This novel finding refines our understanding of individual differences in EL’s relational effects, providing a nuanced perspective on SE’s moderating role.

### 5.2 Practical implications

The empirical findings of this study offer important practical implications for fostering positive marital attitudes and facilitating healthy intimate relationship development among young adults in contemporary society, particularly amid the pervasive emotional labor demands of service-oriented economies and declining marital intentions.

First, targeted interventions for adaptive emotional labor (EL) management are essential to mitigate the depletion of young adults’ psychological resources. Specifically, workplace training and social skill development programs should prioritize equipping young adults with adaptive emotional regulation strategies—emphasizing deep acting (Kim and Oh, 2023) (i.e., modifying internal emotional states to align with required displays) over surface acting (i.e., merely adjusting external emotional expressions without internal congruence). This approach can reduce the sense of inauthenticity and emotional exhaustion associated with EL (Fatima et al., 2018), as deep acting is less resource-depleting and more sustainable than surface acting (Grandey, 2003). By preserving psychological resources for investment in intimate relationship development and marital decision-making, such interventions can effectively alleviate the negative cross-domain spillover of EL on marital intentions (MI), aligning with the Conservation of Resources Theory framework validated in our findings (Hu et al., 2023).

Second, cognitive restructuring interventions targeting discrepancy feelings (DF) can help young adults align their marital expectations with realistic experiences (Xie and Hong, 2022). Counselors and relationship educators should guide young adults to recognize how EL-induced DF generalize to their appraisals of marriage, challenge overly idealized marital expectations, and cultivate a more realistic, flexible perception of conjugal life. For instance, interventions can help individuals distinguish between workplace emotional performance and authentic emotional expression in intimate relationships, reducing the misalignment between ideal marital emotional states and anticipated reality. This cognitive adjustment directly disrupts the mediating pathway of EL → DF → MI, mitigating the adverse impact of perceived marital discrepancies on MI as identified in our mediation analysis (Yuan et al., 2022).

Third, self-esteem (SE) enhancement interventions should be tailored to the specific moderating role of SE observed in this study, with targeted strategies for different SE levels. For young adults with high SE (Cerolini et al., 2023; Ibrahim et al., 2025)—who exhibited a stronger direct negative effect of EL on MI—psychological counseling and positive psychology interventions should focus on building a more flexible self-concept, reducing sensitivity to EL-induced inauthenticity, and buffering the amplified adverse impact of EL on MI. For those with low SE (Chen et al., 2020), interventions should prioritize improving self-concept clarity and emotional awareness, helping them recognize the potential negative effects of EL on their intimate relationship attitudes—even though the direct EL → MI link was weaker in this group. Such tailored interventions acknowledge the nuanced moderating role of SE, maximizing their effectiveness in regulating the EL–MI relationship.

Additionally, the findings provide valuable insights for designing family and relationship education programs for young adults. Educators and counselors should integrate content on the spillover effects of EL into relationship education curricula (Dobrowolska et al., 2020), helping young adults identify how EL in work and social interactions shapes their marital attitudes. These programs can further teach practical skills for managing emotional strain (Rübben, 2025), adjusting marital expectations, and building adaptive SE—skills that are increasingly critical in modern service-oriented societies, where young adults face mounting EL demands in both professional and interpersonal contexts. By proactively addressing EL-related psychological challenges, such programs can foster healthier marital attitudes and support positive relationship formation among contemporary youth.

Notably, the present model demonstrated relatively small effect sizes in predicting young adults’ marital and fertility intentions (R^2^ = 0.030 for the final model predicting marital intentions). While the low explanatory power of the model is a methodological limitation that should be acknowledged, these small effect sizes also carry meaningful practical implications for understanding contemporary young people’s marriage intentions in the digital age. The modest predictive power suggests that young adults’ decisions regarding marriage and childbearing are shaped by a complex constellation of factors beyond the scope of the current investigation, including broader socioeconomic conditions, cultural shifts, and personal life trajectories. This aligns with the observation that marriage is increasingly viewed as a voluntary and deliberative choice rather than a normative life stage, making it less susceptible to prediction based on a limited set of psychological and relational variables. The experience of emotional and behavioral discrepancies in virtual intimate relationships, and the way self-esteem moderates these experiences (as indicated by the significant X × W interaction term, b = −0.66, *p* < 0.01), appear to subtly shape young people’s perceptions of marriage as a viable and desirable life option. In this sense, the findings underscore that even seemingly minor shifts in relational experiences and self-perceptions can accumulate to influence major life decisions, particularly in a context where young people are navigating increasingly fluid and technologically mediated forms of intimacy. The small effect sizes reflect the nuanced and context-dependent nature of marriage intentions in contemporary society. For researchers and practitioners, these findings suggest that interventions aimed at promoting healthy marital and family formation among young adults should not focus on isolated psychological factors, but should instead adopt a holistic approach that accounts for the multifaceted and evolving nature of young people’s intimate lives.

### 5.3 Limitations and future directions

Despite its theoretical and practical contributions, this study has three key limitations that suggest directions for future research. First, the cross-sectional design only permits the examination of correlational relationships among emotional labor (EL) (Kim and Park, 2023), discrepancy feelings (DF) (Lu et al., 2019), self-esteem (SE), and marital intentions (MI) (Yuan et al., 2022), which prevents causal inferences (Kennedy and Arendale, 2023). The reverse causal pathway—such as low MI leading to more frequent engagement in EL—cannot be ruled out. Future longitudinal research should track these variables over time to clarify causal directions and explore the long-term spillover effects of EL (Lyons et al., 2023) on marital decision-making (Shen et al., 2023).

Second, the exclusive reliance on self-report measures for all core variables may introduce common method variance and social desirability bias (Blair et al., 2025). For instance, participants may under-report emotional labor (EL) or discrepancy feelings (DF) due to concerns about social approval. Future studies are encouraged to adopt multi-method and multi-informant approaches, such as partner reports, behavioral observations, or situational experiments, to reduce measurement bias (Gavazzi et al., 2023) and enhance data validity (Lajunen and Gaygısız, 2019). In addition, the sample consisting of Chinese young adults limits cross-cultural generalization (Bonifacci et al., 2023). Replication of the model in Western and other non-Chinese populations would help verify its cross-cultural validity and identify potential cultural moderators, such as individualism–collectivism (Lin et al., 2025).

Third, the current framework has limitations in explanatory scope. It only considers DF as a mediator, ignores EL subtypes (surface acting vs. deep acting) and contextual differences (work vs. social settings), and shows relatively low explanatory power for the variance in DF and MI (R^2^ = 0.02 and 0.030, respectively). Future research could build a multiple mediation model including variables such as emotional exhaustion or fear of intimacy, distinguish EL by type and context to explore their differential effects, and integrate more factors (e.g., family relationship quality, neuroticism) to improve the model’s explanatory power and comprehensiveness.

## 6 Conclusion

The present study empirically validates a moderated mediation model among Chinese young adults: emotional labor (EL) exerts a negative impact on young adults’ marital intentions (MI) through the partial mediating role of discrepancy feelings (DF), while self-esteem (SE) significantly moderates the direct negative relationship between EL and MI. Specifically, the direct adverse effect of EL on MI is more pronounced among individuals with high SE and weaker among those with low SE. Notably, the indirect effect of EL on MI via DF remains statistically significant across all SE levels and is not moderated by SE, confirming the stability of this cognitive mediating pathway.

These findings advance our theoretical understanding of the psychological mechanisms and boundary conditions that link emotional labor (EL) (Lin et al., 2025) to young adults’ marital intentions (MI). By extending the cross-domain spillover effects of EL to the realm of intimate relationship attitudes, the study enriches the existing literature on EL’s consequences beyond workplace and general well-being outcomes. It further clarifies how cognitive factors (i.e., DF) and personality traits (i.e., SE) jointly shape the EL-MI relationship, refining the theoretical framework integrating Conservation of Resources Theory and Cognitive Dissonance Theory. Practically, the results underscore that targeted interventions—focusing on adaptive EL management (Rodrigues et al., 2025), cognitive restructuring of DF, and tailored SE enhancement—can effectively mitigate EL’s negative impact on MI, thereby fostering positive marital attitudes and healthy intimate relationship development among young adults.

In summary, this study highlights the pivotal role of daily EL experiences in shaping young adults’ marital decision-making processes. Despite its inherent limitations (e.g., cross-sectional design, single-source data), the findings provide a valuable theoretical and empirical foundation for understanding and promoting young adults’ marital well-being in contemporary society, where demands for EL are increasingly pervasive. Future research addressing the identified limitations will further refine our comprehension of this complex relationship, supporting more targeted strategies to address declining marital intentions among young adults.

## Data Availability

The datasets presented in this study can be found in online repositories. The names of the repository/repositories and accession number(s) can be found in the article/supplementary material.
