# Correction: How emotional labor shapes marital intentions: a moderated mediation model of discrepancy feelings and self-esteem in young adults

**DOI:** 10.3389/fpsyg.2026.1832864

**Published:** 2026-03-30

**Authors:** Frontiers Production Office

**Affiliations:** Frontiers Media SA, Lausanne, Switzerland

**Keywords:** discrepancy feelings, emotional labor, marital intentions, moderated mediation, self-esteem, young adults

Affiliation Educational and Psychological College, Hubei Engineering University, Xiaogan, China was omitted for author Yuxia Luo. This affiliation has now been added for author Yuxia Luo.

The original version of this article has been updated.

